# Development and validation of a predictive mortality scoring model for bloodstream infections due to *Escherichia coli* in the PROBAC cohort

**DOI:** 10.1007/s15010-025-02614-9

**Published:** 2025-07-23

**Authors:** Paula Olivares-Navarro, María Teresa Pérez-Rodríguez, Ane Josune Goikoetxea-Agirre, José María Reguera-Iglesias, Eva León, María Ángeles Mantecón, Ángeles Pulido-Navazo, Lucía Boix-Palop, Pilar Retamar-Gentil, Carlos Armiñanzas-Castillo, Isabel Fernández-Natal, Alfredo Jover-Sáenz, Alfonso del Arco Jiménez, Jonathan Fernández-Suárez, Andrés Martín-Aspas, Alejandro Smithson-Amat, Alberto Bahamonde Carrasco, Clara Natera-Kindelán, Pedro Martínez Pérez-Crespo, Inmaculada López Hernández, Luis Eduardo López-Cortés, Jesús Rodríguez-Baño

**Affiliations:** 1 Unidad Clínica de Enfermedades Infecciosas y Microbiología, Hospital Universitario Virgen Macarena, Avda Dr. Fedriani, 3, 41009 Seville, Spain; 2 CIBERINFEC, Instituto de Salud Carlos III, Madrid, Spain; 3 Complejo Hospitalario Universitario de Vigo, Galicia Sur Health Research Institute, Vigo, Spain; 4 Servicio de Enfermedades Infecciosas, Hospital Universitario de Cruces, Bilbao, Spain; 5 Hospital Regional Universitario de Málaga, IBIMA, Málaga, Spain; 6 Servicio de Enfermedades Infecciosas, Hospital Universitario Virgen de Valme, Seville, Spain; 7 Servicio de Enfermedades Infecciosas, Hospital Universitario de Burgos, Burgos, Spain; 8 Servicio de Enfermedades Infecciosas, Hospital General de Granollers, Granollers, Spain; 9 Servicio de Enfermedades Infecciosas, Hospital Universitario Mútua de Terrassa, Terrassa, Spain; 10 Servicio de Enfermedades Infecciosas, Hospital Universitario Marqués de Valdecilla, Santander, Spain; 11 Servicio de Microbiología Clínica, Complejo Asistencial Universitario de León, León, Spain; 12 Unidad Territorial Infección Nosocomial y Política Antibiótica (UTIN), Hospital Universitario Arnau de Vilanova, Lleida, Spain; 13 Grupo de Enfermedades Infecciosas, Servicio de Medicina Interna, Hospital Universitario Costa del Sol, Marbella, Spain; 14 Servicio de Enfermedades Infecciosas, Hospital Universitario Central de Asturias, Oviedo, Spain; 15 Facultad de Medicina, Instituto de Investigación E Innovación en Ciencias Biomédicas de Cádiz (INiBICA), Unidad de Enfermedades Infecciosas, Servicio de Medicina InternaHospital Universitario Puerta del MarUniversidad de Cádiz, Cadiz, Spain; 16 Unidad de Medicina Interna, Hospital Esperit Sant, Santa Coloma de Gramenet, Barcelona, Spain; 17 Departamento de Medicina Interna, Hospital de El Bierzo, Ponferrada, Spain; 18 Servicio de Enfermedades Infecciosas, Hospital Universitario Reina Sofia, Córdoba, Spain; 19 Departamento de Medicina, Universidad de Sevilla, Instituto de Biomedicina de Sevilla (IBiS)/CSIC, Seville, Spain

**Keywords:** Predictive model, Bloodstream infection, Mortality, *Escherichia coli*

## Abstract

**Introduction:**

*Escherichia coli* is the most frequent cause of bacteraemia and has a major impact on morbidity and mortality. The aim of this study is to define and internally validate a predictive risk score of 30-day all-cause mortality.

**Methods:**

A prospective, multicentre, cohort study conducted in 26 Spanish hospitals between October 2016 and March 2017 was performed. All monomicrobial *E. coli* bloodstream infections (BSIs) were included. The primary outcome was 30-day all-cause mortality. Cases were randomized to a derivation cohort (DC) and a validation cohort (VC). The predictive score was calculated from a multivariable model performed by logistic regression in the DC and subsequently applied to the VC. The predictive ability of the model was estimated by calculating the area under the ROC curve (AUROC) and the goodness of fit by Hosmer–Lemeshow test and calibration plot.

**Results:**

Overall, 1435 cases were included in the DC and 715 in the VC. The final multivariable model for mortality in DC included (adjusted OR; 95% CI) age over 55 years (2.10; 1.01–4.36), dementia (2.08; 1.24–3.50), liver disease (1.81; 0.99–3.28), healthcare-associated acquisition (2.29; 1.52–3.44), Pitt index > 3 (3.59; 2.30–5.61), SOFA ≥ 2 (1.66; 1.04–2.64), and urinary tract source (0.37; 0.24–0.56). The predictive score showed an AUROC of 0.78 (95% CI 0.74–0.83) in the DC and 0.78 (95% CI 0.73–0.84) in the VC.

**Conclusion:**

We developed and internally validated a predictive scoring model to identify patients with *E. coli* bacteraemia at high and low risk of crude mortality on day 30 of BSI.

**Supplementary Information:**

The online version contains supplementary material available at 10.1007/s15010-025-02614-9.

## Introduction

*Escherichia coli* is the most common cause of bacterial bloodstream infections (BSIs) in high-income countries [1–3]. The increased incidence with increasing antimicrobial resistance makes it one of the microorganisms with the highest burden of mortality in Europe [4]. *Escherichia coli* BSIs are characterized as community acquisition and urinary source. These factors are usually associated with a low risk of mortality, but this is not always true, as other factors such as the patient’s features, time to appropriate treatment or the severity of the presentation of the infection have a major influence on mortality. For this reason, the identification of risk factors associated with *E. coli* mortality and the development of a predictive model is necessary and could be very useful.

In the literature there are several prognostic scores developed specifically for patients with BSIs, but all of them were developed for aetiologies (Gram negative [5] or extended-spectrum β-lactamases (ESBL) producing *Enterobacteriaceae* score [6]) or BSIs in general [7, 8], but not for specific microorganisms. Furthermore, in most of these scores, the risk of *E. coli* is often underestimated when compared to aetiologies or characteristics associated with higher mortality. For example, in the PROBAC score [7], one of the variables in the scoring model is high-risk aetiology and only *Staphylococcus aureus*, *Pseudomonas aeruginosa*, *Acinetobacter baumannii*, *Serratia marcescens*, *Enterococcus* spp. and *Listeria monocytogenes* were included in this category.

The aim of this study was to develop and internally validate a score model to estimate the risk of 30-day all-cause mortality in patients with BSIs due to *E. coli.*

## Methods

### Study design and participants

This analysis is part of the PROBAC project, a national, multicentre, prospective cohort study of microbiologically confirmed bloodstream infection (BSI) in patients aged 14 years or older admitted in 26 participating Spanish hospitals between October 2016 and March 2017 (ClinicalTrials.gov identifier: NCT03148769). The design and methods of the PROBAC study were previously detailed [9]. In summary, study participants were identified by daily review of blood culture results at each hospital and followed for 30 days after the blood cultures were obtained. Data collection was performed directly from the patients’ medical records or by interviewing the patients by previously trained investigators supervised by infectious diseases specialist from each participant site. All recorded data were anonymised. Blood cultures were collected, processed, and interpreted according to Spanish recommendations [10].

For this analysis, all patients with monomicrobial BSIs due to *E. coli* included in the PROBAC database were eligible. Because the predictive score was thought to be used once the identification of *E. coli* in blood cultures is available, patients who died in the first 24 h were excluded; we also excluded patients for whom limitation of therapeutic effort had been previously decided. Patients included were randomly assigned to a derivation cohort (DC; two-thirds of the patients) and the rest to a validation cohort (VC; one-third), using the SPSS random selection tool.

### Variables and definitions

The outcome variable was all-cause mortality at day 30. Independent variables were selected according to previous consensus [11] and included demographics (sex, age in years, hospital facility in which the patient was admitted, ward in charge of the admission), chronic underlying diseases, Charlson comorbidity index [12] accounting for age, exposure to invasive procedures and devices 48 h previous to infection (venous catheter, urinary catheter or mechanical ventilation) or during the previous month (surgery), antibiotics received in the previous month, type of infection acquisition (nosocomial, community-onset but healthcare associated, and community [13]), BSI source (urinary tract, biliary tract, non-biliary intra-abdominal, respiratory tract, pneumonia, vascular, skin and soft tissue, others and unknow) according to clinical and microbiological data [14], Pitt [15] and SOFA score [16], antibiotic therapy, i*n vitro* susceptibility of the causative isolate and production of extended-spectrum β-lactamases (ESBL) or carbapenemases as identified by local laboratories.

Missing data were tabulated because the proportion of missing data was low, the analysis was performed without imputation. The total number of patients included in the multivariable analysis is provided.

### Statistical analysis

Patients in DC and VC were described using absolute numbers and proportions for categorical variables, and median values with interquartile range (IQR) for continuous variables including Pitt score, SOFA score and Charlson comorbidity index. A predictive score was calculated using the DC. For that, bivariate analysis of exposures included in Table 1 and mortality were performed using Chi-square or Fisher’s exact test, as appropriate, and odds ratios (ORs) with 95% confidence intervals (CIs) were calculated. Two-sided *p* values were used and correction for multiple testing was not necessary. Continuous variables such as age, Charlson comorbidity index, Pitt score and SOFA score were dichotomized according to mortality in their strata. BSI acquisition was also dichotomized into community and nosocomial/healthcare-associated, considering the mortality of these categories. Because the objective of the score was only to be predictive, no specific attempt to hypothesize the causative relation among variables was attempted. Variables with a bivariate *p* value ≤ 0.2 were included in the multivariable logistic regression model and selected manually using a backward stepwise method by deleting the variable with lower significance (i.e., *p* value closer to 1) at each step, until the most parsimonious model was reached. Effect modification of adequate empirical treatment and BSI source, Pitt and SOFA scores, and the production of ESBL were explored. The discriminatory power of this model was evaluated calculating the area under the empirical receiver operating characteristic curve (AUROC) with 95% CI and the goodness of fit by Hosmer–Lemeshow test and calibration plot.

**Table 1:** Demographics, clinical and epidemiological characteristics of patients in the derivation and validation cohort

Variable	Derivation cohort (*n* = 1639)	Validation cohort (*n* = 819)	*P* value
Median age in years (IQR)	74 (62–82)	73 (61–83)	0.958
Male sex	817 (50.2)	405 (49.8)	0.830
Underlying diseases			
Myocardial infarction	124 (7.6)	60 (7.3)	0.832
Congestive heart failure	179 (10.9)	91 (11.1)	0.887
Cerebrovascular disease	179 (10.9)	74 (9.0)	0.147
Dementia	171 (10.4)	89 (10.9)	0.742
Chronic pulmonary disease	190 (11.6)	97 (11.8)	0.855
Liver disease	110 (6.7)	58 (7.1)	0.732
Diabetes mellitus	418 (25.5)	214 (26.1)	0.738
Moderate or severe kidney	221 (13.5)	99 (12.1)	0.332
Cancer	421 (25.7)	210 (25.7)	0.981
Haematological malignancy	78 (4.8)	41 (5.0)	0.788
Neutrophils < 500 cells/mm^3^	44 (2.7)	25 (3.1)	0.603
Obstructive uropathy	113 (6.9)	46 (5.6)	0.225
Recurrent UTI	158 (9.6)	72 (8.8)	0.496
Obstructive biliary pathology	100 (6.1)	53 (6.5)	0.720
Median Charlson comorbidity index^a^ (IQR)	4 (3–6)	4 (2–6)	0.635
Invasive procedures /devices			
Central venous catheter	135 (8.2)	67 (8.2)	0.962
Urinary catheter	204 (12.5)	96 (11.7)	0.605
Previous surgery	125 (7.6)	60 (7.3)	0.790
Mechanical ventilation	29 (1.8)	11 (1.3)	0.431
BSI acquisition			
Community	867 (53.1)	448 (54.8)	0.424
Healthcare-associated	467 (28.6)	235 (28.7)	0.939
Nosocomial	300 (18.4)	135 (16.5)	0.257
Source			
Urinary tract	973 (59.6)	492 (60.3)	0.736
Biliary tract	308 (18.9)	168 (20.6)	0.309
Non- biliary intra- abdominal	123 (7.5)	57 (7.0)	0.625
Unknow	129 (7.9)	52 (6.4)	0.173
Respiratory tract	33 (2.0)	14 (1.7)	0.604
Vascular	24 (1.5)	7 (0.9)	0.202
Skin and soft tissue	14 (0.9)	11 (1.4)	0.255
Pneumonia	11 (0.7)	8 (1.0)	0.415
Others^b^	24 (1.5)	10 (1.2)	0.571
ESBL production	215 (13.1)	114 (13.9)	0.582
Median Pitt score (IQR)	1 (0–2)	1 (0–2)	0.643
Median SOFA score (IQR)	2 (0–4)	2 (0–4)	0.596
Previous antimicrobials	430 (26.2)	196 (23.9)	0.217
Appropriate empirical therapy	1362 (83.1)	675 (82.4)	0.672
30-day crude mortality	135 (8.2)	68 (8.3)	0.955

*n* Number of patients, *IQR* Interquartile Range, *UTI* Urinary Tract Infection, *BSI* Bloodstream infection, *ESBL* Extended-spectrum β-lactamases, *SOFA* Sequential Organ Failure Assessment

^a^Adjusted for age

^b^Includes central nervous system, osteoarticular and others.

Missing data were included in the supplementary material (Supplementary Table S2)

A predictive score was calculated using the regression coefficients as in Sullivan’s scoring system [17] (ie, dividing the beta regression coefficients in the final multivariable model by half of the smallest value and rounding to the nearest unit). The score was applied to the DC to check its AUROC and goodness of fit. The positive and negative predictive values (PPV, NPV), sensitivity (SE), specificity (SP), accuracy (AC) and positive and negative likelihood ratio (PLR, NLR) of the score were calculated with 95% CI. Then, the score was applied to the VC for internal validation. No specific threshold was searched, as the choice may vary depending on the clinical context and the investigator’s objectives.

In addition, the impact of ESBL production was studied. For this, the model was applied exclusively to this subgroup of patients, and the discriminative power and goodness of fit were once again evaluated using the AUROC and the Hosmer–Lemeshow test.

Finally, our prediction model was compared with previous severity scores such as Pitt score [15] and SOFA score [16], as well as with mortality scoring models described in the literature for Gram-negative bacteria [5] and BSI [7]. For this purpose, all scoring models were applied to our database and the AUROC and goodness of fit were calculated.

All analyses were performed using the SPSS software (IBM Statistics for Windows, v.25.0, IBM Corporation, Armonk, NY, USA); while the calibration plot and discriminative ability of scores was obtained with R software (R Core Team, v4.3.3; R Foundation for Statistical Computing, Vienna, AT, EU) and RStudio software (RStudio, v2025.05.1 + 513, PBC, Boston, MA, USA). The other graphics were created with Microsoft Excel (Microsoft Corporation, v2407, Redmond, WA, USA).

This study was reported according to TRIPOD-ID guidelines [18]. The TRIPOD-ID checklist is shown in Supplementary Table S1.

## Results

The PROBAC cohort included 2520 cases of monomicrobial *E. coli* BSI; 62 patients were excluded, 60 who died in the first 24 h and a further 2 who were under limitation of therapeutic effort. Therefore, 2458 patients were included. Of the cases included, 1639 were assigned to the DC, and 819 to the VC (Supplementary Figure S1). No significant differences in the distribution of variables and cases across centres between the two cohorts were found (centres shown in Supplementary Table S3). The median age of patients in the DC was 74 years (62–82 years); 817 (50.2%) were males. The most frequent sources of BSI were the urinary tract (*n* = 973, 59.4%) and the biliary tract (*n* = 308, 18.8%) and the most frequent acquisition the community (*n* = 867, 53.1%). The features of patients in the VC were similar. All demographics, clinical and epidemiological characteristics of patients in each cohort are shown in Table 1.

**Fig. 1:**
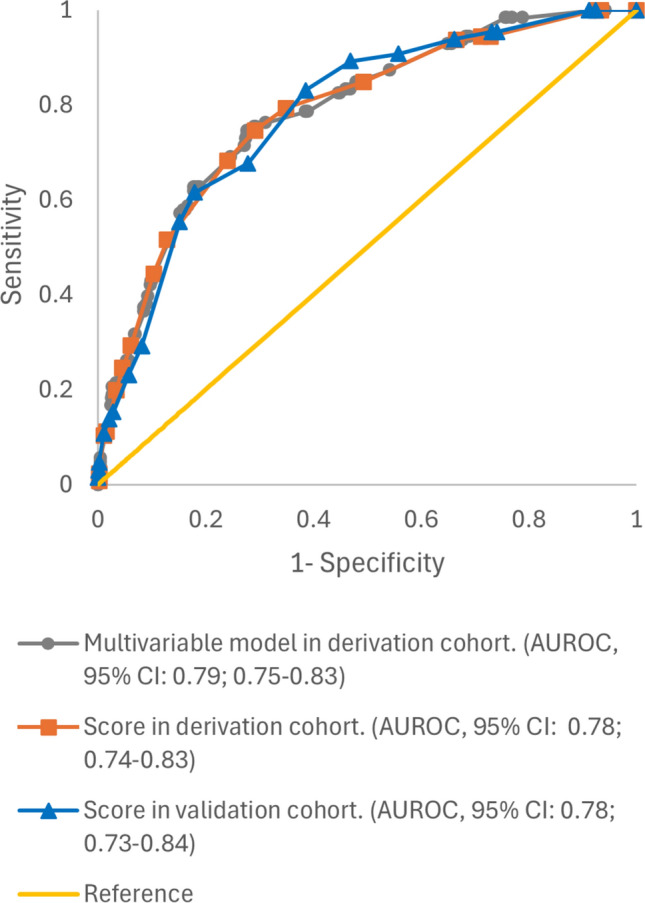
Receiver operating curves for the multivariable model and scoring system in the derivation cohort and validation cohort with their corresponding AUROC and 95% confidence intervals

**Fig. 2:**
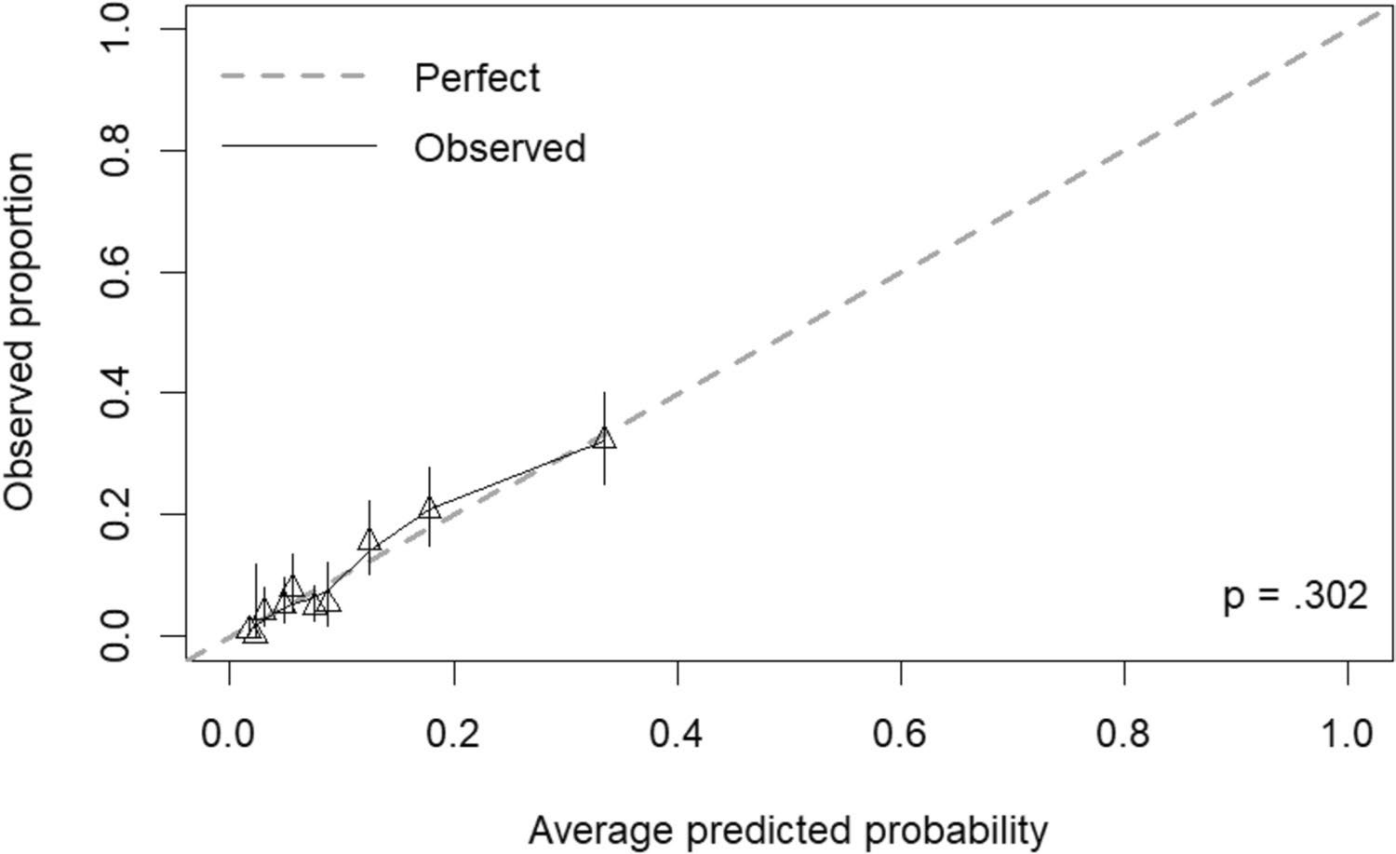
Calibration plot of final multivariable logistic regression model of 30-day mortality in the derivation cohort. *P* value is for comparison of observed and perfect lines

Categorization of continuous and polychotomous variables was as follows: age, ≤ 55 years and > 55 years; Charlson index, ≤ 3 and > 3; Pitt score, ≤ 3 and > 3; SOFA score, < 2 and ≥ 2; and BSI acquisition: healthcare-associated (that included nosocomial and community-onset but healthcare associated) and community (the evaluation of other cohort cut-off points was included in the Supplementary Table S4). The bivariate analysis of the association of clinical and epidemiological variables with mortality at day 30 in the DC cohort is shown in Supplementary Table S3. The variables included in the initial multivariable model were: age > 55 years, male sex, myocardial infarction, dementia, liver disease, neutrophils counts < 500 cells/mm^3^, BSI acquisition, Pitt score > 3; SOFA score ≥ 2, the presence of invasive devices as central venous catheter, urinary catheter and mechanical ventilation; antimicrobials use 30 days prior to infection, urinary tract source and ESBL production in the *E. coli* isolate. The interactions studied were not significant (Supplementary Table S6). The final multivariable model (shown in Table 2) included age > 55 years, dementia, liver disease, healthcare-associated BSI acquisition, urinary tract source, Pitt score > 3 and SOFA score ≥ 2 as mortality predictors. The AUROC of the model was 0.79 (95% CI 0.75–0.83) (Fig. 1) and the Hosmer–Lemeshow test showed a *p* value of 0.57. The calibration plot r showed a good concordance between predicted and observed mortality (Fig. 2).

**Table 2:** Univariable and multivariable analysis of risk factors associated to all-cause 30-day mortality in the derivation cohort, with the deduced score points for each risk factor

Variable	Crude analysis				Adjusted analysis			
	No. deceased (%) [*n* = 135]	No. alive (%) [*n* = 1504]	OR (95% CI)	*P* value	β regression coefficient	OR (95% CI)	*P* value	Score points
Age > 55 years	120 (89.9)	1237 (82.2)	2.60 (1.30–5.19)	0.005	0.74	2.10 (1.01–4.36)	0.048	3
Male sex	82 (60.7)	735 (48.9)	1.66 (1.15–2.40)	0.006				
Dementia	26 (19.3)	145 (9.6)	2.24 (1.41–3.54)	< 0.001	0.73	2.08 (1.24–3.50)	0.006	3
Myocardial infarction	14 (10.4)	110 (7.3)	1.47 (0.82–2.64)	0.198				
Liver disease	20 (14.8)	90 (6.0)	2.73 (1.62–4.60)	< 0.001	0.59	1.81 (0.99–3.28)	0.052	2
Neutrophils < 500 cells/mm^3^	7 (5.2)	37 (2.5)	2.17 (0.95–4.96)	0.061				
Central venous catheter	20 (14.8)	115 (7.6)	2.10 (1.13–3.50)	0.004				
Urinary catheter	22 (16.3)	182 (12.1)	1.41 (0.87–2.29)	0.157				
Mechanical ventilation	5 (3.7)	24 (1.6)	2.37 (0.89–6.32)	0.084				
Health-care acquisition	93 (68.9)	679 (45.1)	2.69 (1.84–3.93)	< 0.001	0.83	2.29 (1.52–3.44)	< 0.001	3
Pitt score > 3	51 (37.8)	164 (10.9)	5.08 (3.46–7.48)	< 0.001	1.28	3.59 (2.30–5.61)	< 0.001	5
SOFA score ≥ 2	105 (77.8)	758 (50.4)	3.44 (2.27–5.23)	< 0.001	0.51	1.66 (1.04–2.64)	0.033	2
Urinary source	46 (34.1)	927 (61.6)	0.32 (0.22–0.47)	< 0.001	-1.00	0.37 (0.24–0.56)	< 0.001	-4
ESBL production	24 (17.8)	191 (12.7)	1.49 (0.93–2.37)	0.094				
Previous antimicrobials	50 (37.0)	380 (25.3)	1.74 (1.20–2.51)	0.003				

*No* Number, *n* Number of patients, *OR* Odds Ratio, *CI* Confidence Interval, *SOFA* Sequential Organ Failure Assessment, *ESBL* Extended-spectrum β-lactamases

The variables selected in multivariable model were transformed into a predictive score (Table 2). The points assigned to patients in the DC cohort ranged from 0 to 16 points. Applying the developed score to DC, the AUROC remains almost unchanged at 0.78 (95% CI 0.74–0.83) (Fig. 1) and the *p* value for the Hosmer–Lemeshow test was 0.35, which also indicated a good fit. The SE, SP, PPV, NPV, PLR, NLR and AC for the different thresholds of the score are shown in Table 3 and with the 95% CI in Supplementary Table S7. A score value ≥ 0 showed an NPV (95% CI) of 98.3% (96.5–99.3%) with a SE (95% CI) of 94.4% (88.9–97.7%); but a low SP (27.2%; 95% CI 24.9–29.5%) and PPV (10.1%; 95% CI 8.4–11.9%). In contrast, a score value ≥ 6 showed a good SP (75.9%; 95% CI 73.6–78.1%) with a NPV (95% CI) of 96.5% (95.3–97.5%).

**Table 3:** Risk score performance in the derivation cohort and the validation cohort (data shown are percentages)

Derivation cohort [*n* = 1588]									Validation cohort [*n* = 796]								
Score	No. of patients (Proportion)	SE	SP	PPV	NPV	AC	PLR	NLR	Score	No. of patients (Proportion)	SE	SP	PPV	NPV	AC	PLR	NLR
≥ -4	1588 (100.0)	100.0	0.0	7.9	NaN	7.9	1.00	NaN	≥ – 4	796 (100.0)	100.0	0.0	8.2	NaN	8.2	1.00	NaN
≥ -2	1492 (94.0)	100.0	6.6	8.5	100.0	14.0	1.07	0.00	≥ – 2	741 (93.1)	100.0	7.5	8.8	100.0	15.1	1.08	0.00
≥ -1	1480 (93.2)	100.0	7.4	8.5	100.0	14.7	1.08	0.00	≥ – 1	732 (92.0)	100.0	8.8	8.9	100.0	16.2	1.10	0.00
≥ 0	1184 (74.6)	94.4	27.2	10.1	98.3	32.5	1.30	0.20	≥ 0	604 (75.9)	95.4	25.9	10.3	98.4	31.5	1.29	0.18
≥ 1	1159 (73.0)	94.4	28.9	10.3	98.4	34.1	1.33	0.19	≥ 1	597 (75.0)	95.4	26.8	10.4	98.5	32.4	1.30	0.17
≥ 2	1090 (68.6)	93.7	33.5	10.8	98.4	38.3	1.41	0.19	≥ 1	545 (68.5)	93.8	33.8	11.2	98.4	38.7	1.42	0.18
≥ 3	829 (52.2)	84.9	50.6	12.9	97.5	53.3	1.72	0.30	≥ 3	467 (58.7)	90.8	44.2	12.6	98.2	48.0	1.63	0.21
≥ 4	610 (38.4)	79.4	65.1	16.4	97.3	66.3	2.28	0.32	≥ 4	401 (50.4)	89.2	53.1	14.5	98.2	56.0	1.90	0.20
≥ 5	521 (32.8)	74.6	70.8	18.0	97.0	71.1	2.55	0.36	≥ 5	336 (42.2)	83.1	61.4	16.1	97.6	63.2	2.15	0.28
≥ 6	438 (27.6)	68.3	75.9	19.6	96.5	75.3	2.83	0.42	≥ 6	248 (31.2)	67.7	72.1	17.7	96.2	71.7	2.43	0.45
≥ 7	253 (15.9)	51.6	87.1	25.7	95.4	84.3	4.01	0.56	≥ 7	171 (21.5)	61.5	82.1	23.4	96.0	80.4	3.43	0.47
≥ 8	208 (13.1)	44.4	89.6	26.9	94.9	86.0	4.27	0.62	≥ 8	147 (18.5)	55.4	84.8	24.5	95.5	82.4	3.65	0.53
≥ 9	127 (8.0)	29.4	93.8	29.1	93.9	88.7	4.77	0.75	≥ 9	79 (9.9)	29.2	91.8	24.1	93.6	86.7	3.56	0.77
≥ 10	98 (6.2)	24.6	95.4	31.6	93.6	89.9	5.37	0.79	≥ 10	57 (7.2)	23.1	94.3	26.3	93.2	88.4	4.02	0.82
≥ 11	75 (4.7)	19.8	96.6	33.3	93.3	90.5	5.80	0.83	≥ 11	31 (4.0)	15.4	97.1	32.3	92.8	90.5	5.36	0.87
≥ 12	37 (2.3)	11.1	98.4	37.8	92.8	91.5	7.06	0.90	≥ 12	24 (3.0)	13.8	97.9	37.5	92.7	91.1	6.75	0.88
≥ 13	29 (1.8)	10.3	98.9	44.8	92.8	91.9	9.43	0.91	≥ 13	15 (1.9)	10.8	98.9	46.7	92.6	91.7	9.84	0.90
≥ 14	7 (0.4)	2.4	99.7	42.9	92.2	92.0	8.70	0.98	≥ 14	5 (0.6)	4.6	99.7	60.0	92.2	92.0	16.87	0.96
≥ 15	6 (0.4)	1.6	99.7	33.3	92.2	92.0	5.85	0.99	≥ 15	4 (0.5)	3.1	99.7	50.0	92.0	91.8	11.25	0.97
≥ 16	4 (0.3)	0.8	99.8	25.0	92.1	91.9	3.87	0.99	≥ 16	3 (0.4)	3.1	99.9	66.7	92.1	92.0	22.49	0.97
									≥ 18	1 (0.1)	1.5	100.0	100.0	91.9	92.0	Inf	0.98

*n* Number of patients, *No*. Number, *SE* Sensitivity, *SP* Specificity, *PPV* Positive Predictive Value, *NPV* Negative Predictive Value, *AC* Accuracy, *PLR* Positive Likelihood Ratio, *NLR* Negative Likelihood Ratio, *NaN* Not a Number, *Inf* Infinity

We then applied the score in our VC cohort, and the range of scores was 0–18 points. The AUROC was 0.78 (95% CI 0.73–0.84) (Fig. 1) and the *p* value for the Hosmer–Lemeshow test was 0.15. The SE, SP, PPV, NPV PLR, NLR and AC for the different thresholds of the score are showed in Table 3 and Supplementary Table S8. A cut-off of ≥ 0 showed 95.4% (95% CI 87.1–99.0%) SE and 98.4% (95% CI 95.5–99.7%) NPV and when a cut-off of ≥ 6 was considered, SP (95% CI) was 72,1% (68.7–75.3%) and NPV (95% CI) of 96.2% (94.2–97.6%).

We identified three groups of mortality risk in both cohort: a low-risk group (< 0 points), a medium-risk group (0–5 points) and high-risk group (≥ 6 points). Mortality rates of patients in the DC and VC in the 3 groups were 1.7% and 1.6%; 3.5% and 3.8%; and 19.6% and 17.7%, respectively.

To evaluate the impact of ESBL production on our score, the predictive score model was applied to this subgroup of patients (*n* = 329 patients). When applied, the AUROC was 0.75 (95% CI 0.67–0.83) (Supplementary Figure S2) and the Hosmer–Lemeshow test showed a *p* value of 0.533.

To conclude, we compare our scoring model with previous models. To do so, we validated other predictive models on our database. To apply the Gram-negative bacteria predictive score [5], we only included patients with appropriate empirical treatment (*n* = 2037 patients). The AUROC of this predictive score was 0.73 (95% CI 0.68–0.77) (Supplementary Figure S3) and the Hosmer–Lemeshow test showed a *p* value of 0.177. In the case of the PROBAC score [7], all patients (*n* = 2458) were included and the AUROC was not notably higher (0.81; 95% CI 0.78–0.84), despite including a higher number of variables (Supplementary Figure S4). In contrast, Hosmer–Lemeshow test showed a significative *p* value (0.008). In addition to these specific scoring model, the Pitt score [15] and SOFA score [16] were also validated. In both cases, the full dataset was used without excluding any patients (*n* = 2.520). The AUROC was 0.72 (95% CI 0.69–0.76) (Supplementary Figure S5) for the Pitt score and 0.74 (95% CI 0.71–0.77) (Supplementary Figure S6) for the SOFA score. The Hosmer–Lemeshow test indicated a good fit for both models, with *p* values of 0.373 for the Pitt score and 0.219 for the SOFA score.

## Discussion

In this study, we developed a predictive score for 30-day mortality in patients with BSI due to *E. coli* using a multicentre prospective cohort, designed to be used at bedside when the microorganism is demonstrated in blood cultures (which typically occurs at 24 h nowadays). The model in which the score was based showed a good predictive ability and calibration both in the DC and VC and classified patients into groups with low, medium and high mortality.

The relevance of developing a score on *E. coli* relies on the fact that it is the most frequent cause of bacteraemia [1, 9] for an estimated incidence rate of 48 per 100,000 person-years, with considerably increasing rates with age (reaching > 300 in persons aged 75 to 85 years) [1] with a population-based estimated mortality of 8.5 deaths/100,000 person-years [19]. The fact that relative associated mortality is lower in *E. coli* in comparison to other aetiologies suggests that a specific predictive score may be useful [20, 21].

Mortality scores have been previously developed could be used to provide context for our results. A score model for patients with BSIs due to Gram-negative bacteria receiving appropriate empirical treatment developed by Al Hasan et al. included 4 predictors: liver cirrhosis, source of infection other than urinary tract or central venous and Pitt score 2–3 or > 4 [5]. Our score included additional variables, some of which may be specific for *E. coli* such as age, dementia, healthcare-associated acquisition and SOFA score. Looking at the variables, although these authors also explored dementia in their model, they did not find it to be related to increased mortality. This may be because the average age of patients in the Al Hasan model is lower than in ours. Other differences with our model that may explain the lower prediction ability of the Al Hasan score in our cohort are the fact that the Al Hasan score only included patients receiving appropriate empirical treatment, and the proportion of *E. coli* was only 38% in the derivation cohort, although it was higher on validation cohort (91%) [22].

Reviewing the literature, we also found two other models to BSIs. One of them is the BLOOMY score [8], which was developed for some aetiologies such as *S. aureus, Enterococcus spp, Klebsiella spp, Enterobacter spp, P. aeruginosa, A. baumannii* and *E. coli*. The percentage of BSIs due to *E. coli* was much lower (only 34%) and the mortality rate was measured at 14 days and 6 months, so it cannot be compared with our score. The other is the PROBAC score [7] developed with the full PROBAC cohort. The full PROBAC score had a lower prediction ability and goodness of fit compared to the specific PROBAC-*E. coli* score shown in this article but include a higher number of variables and therefore this specific score would be more practical specifically for *E. coli* bacteraeamia. The score developed also had higher predictive ability compared to SOFA [16] and Pitt [15] scores. When applied only to patients with ESBL-producing isolates, the score also yielded a good predictive ability, although somehow lower than when applied to the whole cohort of *E. coli* cases.

We think that our predictive model can be very useful to classify patients at the time of diagnosis, and make clinical decisions depending on their mortality risk (e.g., deciding the need for a more intensive management and follow-up in high-risk cases) and for the classification of patients in future clinical trials comparing drugs. This study, however, has several limitations. We only included patients older than 14 years, so the results should not be extrapolated to children. The score was only validated internally. Finally, the results may not apply to areas or populations with a different epidemiology of *E. coli* BSI (e.g., children). Some strengths include the high number of cases included, the quality assessment of data by monitoring and the internal validation.

In summary, a predictive score model of all-cause 30-day mortality in patients with BSI due to *E. coli* was developed and internally validated. The score showed a good prediction ability in both the DC and VC.

## Supplementary Information

Below is the link to the electronic supplementary material.

Supplementary file1 (DOCX 232 KB)

## Data Availability

No datasets were generated or analysed during the current study.
