# Supplementary material for: Development and validation of a predictive mortality scoring model for bloodstream infections due to Escherichia coli in the PROBAC cohort
Source: Infection. 2025 Jul 23;53(6):2681–9. doi: 10.1007/s15010-025-02614-9 (PMC12675721; doi:10.1007/s15010-025-02614-9)
Supplement: Supplementary file 1 — Supplementary file1 (DOCX 232 KB) [file 15010_2025_2614_MOESM1_ESM.docx]

**SUPPLEMENTARY MATERIAL**

**TABLE OF CONTENTS**

|  | **Page** |
| --- | --- |
| **Table S1.** TRIPOD checklist version 15. | 2-3 |
| **Table S2**. Missing data. | 4 |
| **Figure S1.** Flow diagram of inclusion of patients. | 5 |
| **Table S3.**  Distribution of patients per centre across the derivation and validation cohorts. | 6 |
| **Table S4.** Thirty-day mortality stratified by intervals of a continuous variable. | 7 |
| **Table S5.** Univariate analysis of risk factors associated with all-cause 30-day mortality in the derivation cohort. | 8 |
| **Table S6.** Interactions assessed in the final multivariable model. | 9 |
| **Table S7.** Risk score with 95% confidence interval performance in the derivation cohort (data shown are percentages). | 10 |
| **Table S8.** Risk score with 95% confidence interval performance in the validation cohort (data shown are percentages). | 11 |
| **Figure S2.** Receiver operating characteristic curve for 30-day mortality based on the *E. coli* score in the subgroup of patients with extended-spectrum β-lactamase production. | 12 |
| **Figure S3.** Receiver operating curve for 30-day mortality of the gram-negative bacteria predictive model in our database. | 13 |
| **Figure S4**. Receiver operating curve for 30-day mortality of PROBAC score in our database. | 14 |
| **Figure S5.** Receiver operating curve for 30-day mortality of the Pitt score in our database. | 15 |
| **Figure S6.** Receiver operating curve for 30-day mortality of the SOFA score in our database. | 16 |

**Table S1**. TRIPOD checklist version 15 [1]

| **Section/Topic** | **Item** |  | **Checklist Item** | **Page** |
| --- | --- | --- | --- | --- |
| **Title and abstract** | | | | |
| Title | 1 | D;V | Identify the study as developing and/or validating a multivariable prediction model, the target population, and the outcome to be predicted. | 1 |
| Abstract | 2 | D;V | Provide a summary of objectives, study design, setting, participants, sample size, predictors, outcome, statistical analysis, results, and conclusions. | 5 |
| **Introduction** | | | | |
| Background and objectives | 3a | D;V | Explain the medical context (including whether diagnostic or prognostic) and rationale for developing or validating the multivariable prediction model, including references to existing models. | 6 |
|  | 3b | D;V | Specify the objectives, including whether the study describes the development or validation of the model or both. | 6 |
| **Methods** | | | | |
| Source of data | 4a | D;V | Describe the study design or source of data (e.g., randomized trial, cohort, or registry data), separately for the development and validation data sets, if applicable. | 7 |
|  | 4b | D;V | Specify the key study dates, including start of accrual; end of accrual; and, if applicable, end of follow-up. | 7 |
| Participants | 5a | D;V | Specify key elements of the study setting (e.g., primary care, secondary care, general population) including number and location of centres. | 7 |
|  | 5b | D;V | Describe eligibility criteria for participants. | 7 |
|  | 5c | D;V | Give details of treatments received, if relevant. | NA |
| Outcome | 6a | D;V | Clearly define the outcome that is predicted by the prediction model, including how and when assessed. | 7 |
|  | 6b | D;V | Report any actions to blind assessment of the outcome to be predicted. | 7 |
| Predictors | 7a | D;V | Clearly define all predictors used in developing or validating the multivariable prediction model, including how and when they were measured. | 8 |
|  | 7b | D;V | Report any actions to blind assessment of predictors for the outcome and other predictors. | 8 |
| Sample size | 8 | D;V | Explain how the study size was arrived at. | NA |
| Missing data | 9 | D;V | Describe how missing data were handled (e.g., complete-case analysis, single imputation, multiple imputation) with details of any imputation method. | 8 |
| Statistical analysis methods | 10a | D | Describe how predictors were handled in the analyses. | 8 |
|  | 10b | D | Specify type of model, all model-building procedures (including any predictor selection), and method for internal validation. | 8,9 |
|  | 10c | V | For validation, describe how the predictions were calculated. | 9 |
|  | 10d | D;V | Specify all measures used to assess model performance and, if relevant, to compare multiple models. | 9 |
|  | 10e | V | Describe any model updating (e.g., recalibration) arising from the validation, if done. | NA |
| Risk groups | 11 | D;V | Provide details on how risk groups were created, if done. | 8,9 |
| Development vs. validation | 12 | V | For validation, identify any differences from the development data in setting, eligibility criteria, outcome, and predictors. | 8,9 |
| **Results** | | | | |
| Participants | 13a | D;V | Describe the flow of participants through the study, including the number of participants with and without the outcome and, if applicable, a summary of the follow-up time. A diagram may be helpful. | 9,10 |
|  | 13b | D;V | Describe the characteristics of the participants (basic demographics, clinical features, available predictors), including the number of participants with missing data for predictors and outcome. | 10, 14 |
|  | 13c | V | For validation, show a comparison with the development data of the distribution of important variables (demographics, predictors and outcome). | 14 |
| Model development | 14a | D | Specify the number of participants and outcome events in each analysis. | 14 |
|  | 14b | D | If done, report the unadjusted association between each candidate predictor and outcome. | 15 |
| Model specification | 15a | D | Present the full prediction model to allow predictions for individuals (i.e., all regression coefficients, and model intercept or baseline survival at a given time point). | 10, 15 |
|  | 15b | D | Explain how to the use the prediction model. | 10 |
| Model performance | 16 | D;V | Report performance measures (with CIs) for the prediction model. | 10, 15 |
| Model-updating | 17 | V | If done, report the results from any model updating (i.e., model specification, model performance). | NA |
| **Discussion** | | | | |
| Limitations | 18 | D;V | Discuss any limitations of the study (such as nonrepresentative sample, few events per predictor, missing data). | 13 |
| Interpretation | 19a | V | For validation, discuss the results with reference to performance in the development data, and any other validation data. | 12, 13 |
|  | 19b | D;V | Give an overall interpretation of the results, considering objectives, limitations, results from similar studies, and other relevant evidence. | 12, 13 |
| Implications | 20 | D;V | Discuss the potential clinical use of the model and implications for future research. | 13 |
| **Other information** | | | | |
| Supplementary information | 21 | D;V | Provide information about the availability of supplementary resources, such as study protocol, Web calculator, and data sets. | 20, 21 |
| Funding | 22 | D;V | Give the source of funding and the role of the funders for the present study. | 4 |
| Derivation, D; Validation, V; Not Applicable, NA | | | | |

| **Table S2**. Missing data | |
| --- | --- |
| Variable | No. of patients with missing data (%) |
| Age | 48 (2.0) |
| Sex | 17 (0.7) |
| Pitt score | 26 (1.1) |
| Number, No. | |

***
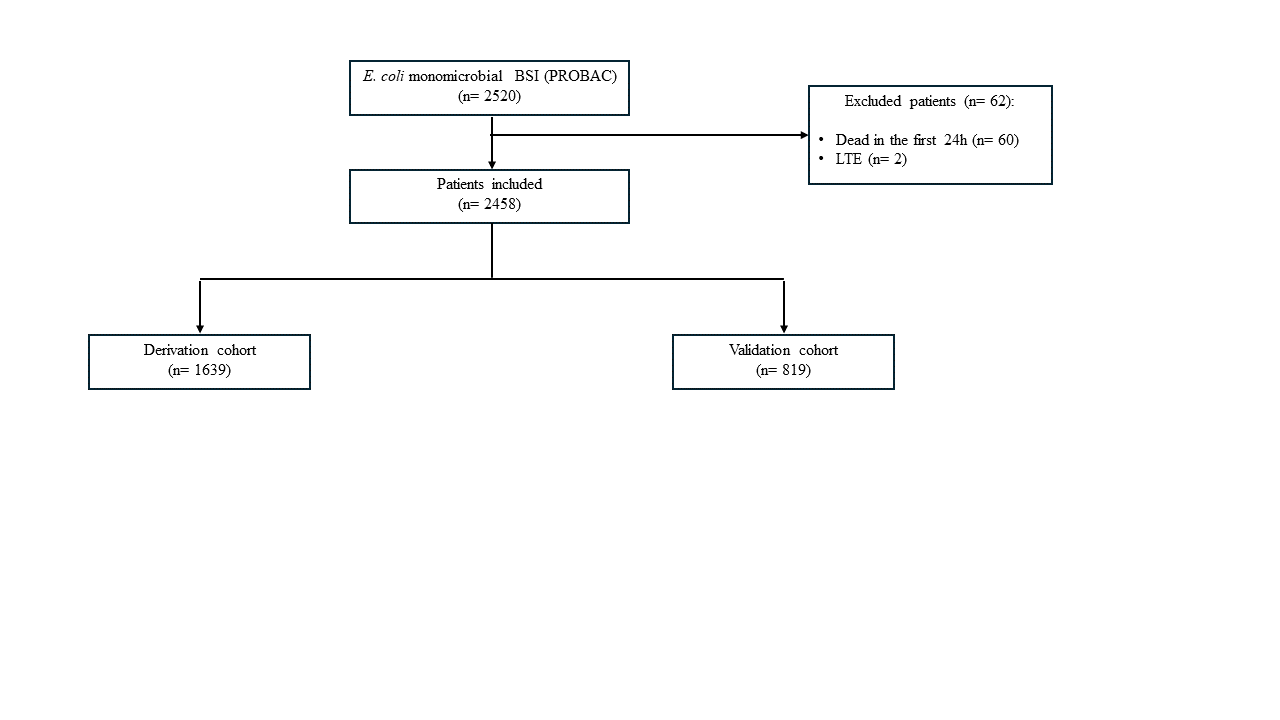
*Figure S1.** Flow diagram of inclusion of patients.

Legend: number of patients, n; Bloodstream infection, BSI; Limitation of Therapeutic Effort, LTE.

| **Table S3.**  Distribution of patients per centre across the derivation and validation cohorts. Frequencies were calculated based on the number of patients of the respective centre. *P* values for the comparison of proportion of patients at each centre between the derivation and validation cohorts were calculated by Chi squared test. Data are no. of patients (percentage within each centre). | | | |
| --- | --- | --- | --- |
| Centre | Derivation cohort  (n= 1639) | Validation cohort  (n= 819) | *P* value |
| Vigo University Hospital Complex | 285 (65.1) | 153 (34.9) | 0.430 |
| Cruces University Hospital | 175 (69.7) | 76 (30.3) | 0.281 |
| Málaga Regional University Hospital | 145 (67.1) | 71 (31.9) | 0.883 |
| Virgen de Valme University Hospital | 112 (70.0) | 48 (30.0) | 0.357 |
| Burgos University Hospital | 104 (71.2) | 42 (28.8) | 0.230 |
| Granollers General Hospital | 85 (64.4) | 47 (35.6) | 0.567 |
| Mútua de Terrassa University Hospital | 79 (63.7) | 45 (36.3) | 0.471 |
| Virgen Macarena University Hospital | 79 (67.5) | 38 (32.5) | 0.843 |
| Marqués de Valdecilla University Hospital | 69 (63.3) | 40 (36.7) | 0.444 |
| León University Healthcare Complex | 55 (59.1) | 38 (40.9) | 0.116 |
| Arnau de Vilanova University Hospital | 56 (64.4) | 31 (35.6) | 0.641 |
| Costa del Sol University Hospital | 53 (63.9) | 30 (36.1) | 0.579 |
| Central University Hospital of Asturias | 54 (70.1) | 23 (29.9) | 0.514 |
| Puerta del Mar University Hospital | 51 (72.9) | 19 (27.1) | 0.266 |
| Esperit Sant Hospital | 44 (72.1) | 17 (27.9) | 0.360 |
| El Bierzo Hospital | 32 (64.0) | 18 (36.0) | 0.685 |
| Reina Sofía University Hospital | 29 (74.4) | 10 (25.6) | 0.305 |
| La Línea University Hospital | 25 (67.6) | 12 (32.4) | 0.908 |
| Jeréz University Hospital | 21 (60.0) | 14 (40.0) | 0.398 |
| Torrecárdenas University Hospital | 21 (65.6) | 11 (34.4) | 0.899 |
| Jaén University Hospital | 16 (61.5) | 10 (38.5) | 0.576 |
| San Cecilio Clinical Hospital | 15 (60.0) | 10 (40.0) | 0.476 |
| Poniente University Hospital | 13 (68.4) | 6 (31.6) | 0.872 |
| San Juan de la Cruz Hospital | 9 (69.2) | 4 (30.8) | 0.845 |
| Alicante General University Hospital | 8 (72.7) | 3 (27.3) | 0.670 |
| Punta de Europa University Hospital | 4 (57.1) | 3 (42.9) | 0.592 |
| Number of patients, n | | | |

| **Table S4.** Thirty-day mortality stratified by intervals of a continuous variable. Data in the table represent the number of deaths/total number of patients in each interval (percentage). | |
| --- | --- |
| Variable | 30-day mortality (%) |
| Age (in years) | |
| ≤ 15 years | 0/1 (0.0) |
| > 15 and ≤ 25 years | 0/19 (0.0) |
| > 25 and ≤ 35 years | 0/36 (0.0) |
| > 35 and ≤ 45 years | 1/73 (1.4) |
| > 45 and ≤ 55 years | 8/121 (6.6) |
| > 55 and ≤ 65 years | 23/248 (9.3) |
| > 65 and ≤ 75 years | 25/389 (6.4) |
| > 75 and ≤ 85 years | 39/468 (8.3) |
| > 85 years | 33/ 252 (13.1) |
| Charlson comorbidity index^a^ (in points) | |
| ≤ 1 | 11/265 (4.2) |
| > 1 and ≤ 2 | 7/127 (5.5) |
| > 2 and ≤ 3 | 9/183 (4.9) |
| > 3 and ≤ 4 | 20/248 (8.1) |
| > 4 and ≤ 5 | 16/191 (8.4) |
| > 5 and ≤ 6 | 24/219 (11.0) |
| > 6 and ≤ 7 | 18/145 (12.4) |
| > 7 and ≤ 8 | 7/86 (8.1) |
| > 8 | 23/175 (13.1) |
| Pitt score (in points) | |
| ≤ 1 | 50/1076 (4.6) |
| > 1 and ≤ 2 | 15/220 (6.8) |
| > 2 and ≤ 3 | 9/92 (9.8) |
| > 3 and ≤ 4 | 16/109 (14.7) |
| > 4 and ≤ 5 | 10/46 (21.7) |
| > 5 and ≤ 6 | 12/27 (44.4) |
| > 6 | 20/50 (40.0) |
| SOFA score (in points) | |
| < 1 | 20/492 (4.1) |
| ≥ 1 and < 2 | 10/290 (3.4) |
| ≥ 2 and < 3 | 14/225 (6.2) |
| ≥ 3 and < 4 | 15/192 (7.8) |
| ≥ 4 and < 5 | 15/132 (11.4) |
| ≥ 5 | 61/308 (19.8) |
| Sequential Organ Failure Assessment, SOFA.  ^a^Adjusted for age. | |

| **Table S5.** Bivariate analysis of risk factors associated with all-cause 30-day mortality in the derivation cohort. | | | | |
| --- | --- | --- | --- | --- |
| Variable | No. deceased  (%) n= 135 | No. alive  (%) n= 1504 | OR (95% CI) | *P* value |
| Age > 55 years | 120 (88.9) | 1237 (82.2) | 2.60 (1.30 - 5.19) | 0.005 |
| Male sex | 82 (60.7) | 735 (48.9) | 1.66 (1.15 - 2.40) | 0.006 |
| Underlying diseases | | | |  |
| Myocardial infarction | 14 (10.4) | 110 (7.3) | 1.47 (0.82 - 2.64) | 0.198 |
| Congestive heart failure | 16 (11.9) | 163 (10.8) | 1.11 (0.64 - 1.91) | 0.717 |
| Cerebrovascular disease | 18 (13.3) | 161 (10.8) | 1.28 (0.76 - 2.16) | 0.348 |
| Dementia | 26 (19.3) | 145 (9.6) | 2.24 (1.41 - 3.54) | <0.001 |
| Chronic pulmonary disease | 19 (14.1) | 171 (11.3) | 1.28 (0.77 - 2.13) | 0.347 |
| Liver disease | 20 (14.8) | 90 (6.0) | 2.73 (1.62 - 4.60) | <0.001 |
| Diabetes mellitus | 31 (23.0) | 387 (25.7) | 0.86 (0.57 - 1.31) | 0.480 |
| Moderate or severe kidney | 19 (14.1) | 202 (13.4) | 1.06 (0.64 - 1.75) | 0.830 |
| Cancer | 36 (26.7) | 377 (25.0) | 1.09 (0.73 - 1.62) | 0.685 |
| Haematological malignancy | 9 (6.7) | 69 (4.6) | 1.49 (0.73 - 3.05) | 0.277 |
| Neutrophils < 500 cells/mm^3^ | 7 (5.2) | 37 (2.4) | 2.17 (0.95 - 4.96) | 0.061 |
| Obstructive uropathy | 7 (5.2) | 106 (7.0) | 0.72 (0.33 - 1.58) | 0.413 |
| Recurrent UTI | 11 (8.1) | 147 (9.8) | 0.82 (0.43 - 1.56) | 0.540 |
| Obstructive biliary pathology | 10 (7.4) | 90 (6.0) | 1.26 (0.64 - 2.48) | 0.508 |
| Invasive procedures /devices | | | |  |
| Central venous catheter | 20 (14.8) | 115 (7.6) | 2.10 (1.13 - 3.50) | 0.004 |
| Urinary catheter | 22 (16.3) | 182 (12.1) | 1.41 (0.87 - 2.29) | 0.157 |
| Previous surgery | 11 (8.1) | 114 (7.6) | 1.08 (0.57 - 2.06) | 0.812 |
| Mechanical ventilation | 5 (3.7) | 24 (2.0) | 2.37 (0.89 - 6.32) | 0.084 |
| BSI acquisition | | | |  |
| Community | 42 (31.1) | 825 (54.9) | 0.37 (0.25 - 0.54) | <0.001 |
| Healthcare associated | 58 (43.0) | 409 (27.2) | 2.01 (1.40 - 2.88) | <0.001 |
| Nosocomial | 35 (25.9) | 265 (17.6) | 1.63 (1.09 - 2.45) | 0.018 |
| Health-care and nosocomial | 93 (68.9) | 679 (45.1) | 2.69 (1.84 - 3.93) | <0.001 |
| Source | | | |  |
| Urinary tract | 46 (34.1) | 927 (61.6) | 0.32 (0.22 - 0.47) | <0.001 |
| Biliary tract | 30 (22.2) | 278 (18.5) | 1.26 (0.82 - 1.93) | 0.287 |
| Intra-abdominal | 17 (12.6) | 106 (7.0) | 1.90 (1.10 - 3.28) | 0.019 |
| Unknow | 17 (12.6) | 112 (7.4) | 1.79 (1.04 - 3.08) | 0.033 |
| Respiratory tract | 8 (5.9) | 25 (1.7) | 3.73 (1.65 - 8.43) | 0.004* |
| Vascular | 6 (4.4) | 18 (1.1) | 3.84 (1.45 - 9.84) | 0.003 |
| Skin and soft tissue | 3 (2.2) | 11 (0.7) | 3.09 (0.85 - 11.19) | 0.071 |
| Pneumonia | 6 (4.4) | 5 (0.0) | 13.95 (4.20 - 46.31) | <0.001* |
| High-risk source^a^ | 59 (43.7) | 299 (19.9) | 3.13 (2.18 - 4.50) | <0.001 |
| ESBL production | 24 (17.8) | 191 (12.7) | 1.49 (0.93 - 2.37) | 0.094 |
| Pitt score > 3 | 51 (37.8) | 164 (10.9) | 5.08 (3.46 - 7.48) | <0.001 |
| SOFA score ≥ 2 | 105 (77.8) | 758 (50.3) | 3.44 (2.27 - 5.23) | <0.001 |
| Previous antimicrobials | 50 (37.0) | 380 (25.2) | 1.74 (1.20 - 2.51) | 0.003 |
| Inappropriate empirical therapy | 26 (19.3) | 251 (16.7) | 1.19 (0.76 - 1.19) | 0.446 |
| Number, No.; Number of patients, n; Odds Ratio, OR; Confidence Interval, CI; Urinary Tract Infection, UTI; Bloodstream infection, BSI; Extended-spectrum β-lactamases, ESBL; Sequential Organ Failure Assessment, SOFA.  ^a^Includes all source except urinary and biliary source  **p* value calculated with Fisher's exact test | | | | |

| **Table S6.** Interactions assessed in the final multivariable model. The OR and *p* values were obtained using the regression model. | | |
| --- | --- | --- |
| Interaction assessed | OR (95% CI) | *P* value |
| Appropriate empirical treatment with urinary source | 0.80 (0.25 - 2.52) | 0.703 |
| Appropriate empirical treatment with high-risk source^a^ | 1.77 (0.58 - 5.40) | 0.319 |
| Appropriate empirical treatment with Pitt score > 3 | 1.52 (0.42 - 5.46) | 0.519 |
| Appropriate empirical treatment with SOFA score ≥ 2 | 1.26 (0.40 - 4.00) | 0.699 |
| Appropriate empirical treatment with ESBL production | 0.56 (0.16 - 1.92) | 0.355 |
| Odds Ratio, OR; Confidence Interval, CI; Sequential Organ Failure Assessment, SOFA; Extended-spectrum β-lactamases, ESBL  ^a^Includes all source except urinary and biliary source | | |

| Table S7. Risk score with 95% confidence interval performance in the derivation cohort (data shown are percentages) | | | | | | | | | |
| --- | --- | --- | --- | --- | --- | --- | --- | --- | --- |
| Score | No. of patients  (Proportion) | SE  (95% CI) | SP  (95% CI) | PPV  (95% CI) | NPV  (95% CI) | AC  (95% CI) | PLR  (95% CI) | NLR  (95% CI) |  |
| ≥-4 | 1588 (100.0) | 100.0 (97.1-100.0) | 0.0 (0.0-0.3) | 7.9 (6.7-9.4) | NaN (0.0-100.0) | 7.9 (6.7-9.4) | 1.00 (1.00-1.00) | NaN (NaN-NaN) |  |
| ≥-2 | 1492 (94.0) | 100.0 (97.1-100.0) | 6.6 (5.4-8.0) | 8.5 (7.1-10.0) | 100.0 (96.2-100.0) | 14.0 (12.3-15.8) | 1.07 (1.06-1.08) | 0.00 (0.00-NaN) |  |
| ≥-1 | 1480 (93.2) | 100.0 (97.1-100.0) | 7.4 (6.1-8.9) | 8.5 (7.1-10.1) | 100.0 (96.6-100.0) | 14.7 (13.0-16.6) | 1.08 (1.06-1.10) | 0.0 (0.00-NaN) |  |
| ≥0 | 1184 (74.6) | 94.4 (88.9-97.7) | 27.2 (24.9-29.5) | 10.1 (8.4-11.9) | 98.3 (96.5-99.3) | 32.5 (30.2-34.9) | 1.30 (1.23-1.37) | 0.20 (0.10-0.42) |  |
| ≥1 | 1159 (73.0) | 94.4 (89.0-97.7) | 28.9 (26.6-31.3) | 10.3 (8.6-12.2) | 98.4 (96.7-99.3) | 34.1 (31.7-36.5) | 1.33 (1.26-1.40) | 0.19 (0.09-0.40) |  |
| ≥2 | 1090 (68.6) | 93.7 (87.9-97.2) | 33.5 (31.1-36.0) | 10.8 (9.0-12.8) | 98.4 (96.9-99.3) | 38.3 (35.9-40.7) | 1.41 (1.33-1.49) | 0.19 (0.10-0.37) |  |
| ≥3 | 829 (52.2) | 84.9 (77.5-90.7) | 50.6 (48.0-53.2) | 12.9 (10.7-15.4) | 97.5 (96.1-98.5) | 53.3 (50.9-55.8) | 1.72 (1.57-1.88) | 0.30 (0.20-0.45) |  |
| ≥4 | 610 (38.4) | 79.4 (71.3-86.1) | 65.1 (62.6-67.6) | 16.4 (13.5-19.6) | 97.3 (96.1-98.3) | 66.3 (63.9-68.6) | 2.28 (2.03-2.55) | 0.32 (0.22-0.45) |  |
| ≥5 | 521 (32.8) | 74.6 (66.1-81.9) | 70.8 (68.4-73.1) | 18.0 (14.8-21.6) | 97.0 (95.8-97.9) | 71.1 (68.8-73.3) | 2.55 (2.24-2.91) | 0.36 (0.27-0.48) |  |
| ≥6 | 438 (27.6) | 68.3 (59.4-76.3) | 75.9 (73.6-78.1) | 19.6 (16.0-23.7) | 96.5 (95.3-97.5) | 75.3 (73.1-77.4) | 2.83 (2.44-3.29) | 0.42 (0.32-0.54) |  |
| ≥7 | 253 (15.9) | 51.6 (42.5-60.6) | 87.1 (85.3-88.8) | 25.7 (20.4-31.5) | 95.4 (94.2-96.5) | 84.3 (82.4-86.1) | 4.01 (3.23-4.98) | 0.56 (0.46-0.67) |  |
| ≥8 | 208 (13.1) | 44.4 (35.6-53.6) | 89.6 (87.9-91.1) | 26.9 (21.0-33.5) | 94.9 (93.6-96.0) | 86.0 (84.2-87.7) | 4.27 (3.34-5.47) | 0.62 (0.53-0.73) |  |
| ≥9 | 127 (8.0) | 29.4 (21.6-38.1) | 93.8 (92.5-95.0) | 29.1 (21.4-37.9) | 93.9 (92.6-95.1) | 88.7 (87.1-90.2) | 4.77 (3.41-6.68) | 0.75 (0.67-0.84) |  |
| ≥10 | 98 (6.2) | 24.6 (17.4-33.1) | 95.4 (94.2-96.4) | 31.6 (22.6-41.8) | 93.6 (92.3-94.8) | 89.8 (88.2-91.2) | 5.37 (3.65-7.89) | 0.79 (0.71-0.87) |  |
| ≥11 | 75 (4.7) | 19.8 (13.3-27.9) | 96.6 (95.5-97.5) | 33.3 (22.9-45.2) | 93.3 (91.9-94.5) | 90.5 (88.9-91.9) | 5.80 (3.72-9.05) | 0.83 (0.76-0.91) |  |
| ≥12 | 37 (2.3) | 11.1 (6.2-17.9) | 98.4 (97.6-99.0) | 37.8 (22.5-55.2) | 92.8 (91.4-94.0) | 91.5 (90.0-92.8) | 7.06 (3.73-13.38) | 0.90 (0.85-0.96) |  |
| ≥13 | 29 (1.8) | 10.3 (5.6-17.0) | 98.9 (98.2-99.4) | 44.8 (26.4-64.3) | 92.8 (91.4-94.0) | 91.9 (90.4-93.2) | 9.43 (4.64-19.15) | 0.91 (0.85-0.96) |  |
| ≥14 | 7 (0.4) | 2.4 (0.5-6.8) | 99.7 (99.3-99.9) | 42.9 (9.9-81.6) | 92.2 (90.8-93.5) | 92.0 (90.6-93.3) | 8.70 (1.97-38.45) | 0.98 (0.95-1.01) |  |
| ≥15 | 6 (0.4) | 1.6 (0.2-5.7) | 99.7 (99.3-99.9) | 33.3 (4.3-77.7) | 92.2 (90.8-93.5) | 92.0 (90.6-93.3) | 5.85 (1.08-31.61) | 0.99 (0.96-1.01) |  |
| ≥16 | 4 (0.3) | 0.8 (0.0-4.3) | 99.8 (99.4-100.0) | 25.0 (0.6-80.6) | 92.1 (90.7-93.4) | 91.9 (90.5-93.2) | 3.87 (0.41-36.91) | 0.99 (0.98-1.01) |  |
| No., Number; Sensitivity, SE; Confidence Interval, CI; Specificity, SP; Positive Predictive Value, PPV; Negative Predictive Value, NPV; Accuracy, AC; Positive Likelihood Ratio, PLR; Negative Likelihood Ratio, NLR; Not a Number, NaN. | | | | | | | | |  |

| **Table S8**. Risk score with 95% confidence interval performance in the validation cohort (data shown are percentages) | | | | | | | | |
| --- | --- | --- | --- | --- | --- | --- | --- | --- |
| Score | No. of patients  (Proportion) | SE  (95% CI) | SP  (95% CI) | PPV  (95% CI) | NPV  (95% CI) | AC  (95% CI) | PLR  (95% CI) | NLR  (95% CI) |
| ≥-4 | 796 (100.0) | 100.0 (94.5-100.0) | 0.0 (0.0-0.5) | 8.2 (6.4-10.3) | NaN (0.0-100.0) | 8.2 (6.4-10.3) | 1.00 (1.00-1.00) | NaN (NaN, NaN) |
| ≥-2 | 741 (93.1) | 100.0 (94.5-100.0) | 7.5 (5.7-9.7) | 8.8 (6.8-11.0) | 100.0 (93.5-100.0) | 15.1 (12.7-17.8) | 1.08 (1.06-1.10) | 0.00 (0.00-NaN) |
| ≥-1 | 732 (92.0) | 100.0 (94.5-100.0) | 8.8 (6.9-11.2) | 8.9 (6.9-11.2) | 100.0 (94.4-100.0) | 16.2 (13.7-19.0) | 1.10 (1.07-1.12) | 0.00 (0.00-NaN) |
| ≥0 | 604 (75.9) | 95.4 (87.1-99.0) | 25.9 (22.7-29.2) | 10.3 (8.0-13.0) | 98.4 (95.5-99.7) | 31.5 (28.3-34.9) | 1.29 (1.20-1.38) | 0.18 (0.06-0.54) |
| ≥1 | 597 (75.0) | 95.4 (87.1-99.0) | 26.8 (23.6-30.2) | 10.4 (8.1-13.1) | 98.5 (95.7-99.7) | 32.4 (29.2-35.8) | 1.30 (1.22-1.40) | 0.17 (0.06-0.52) |
| ≥2 | 545 (68.5) | 93.8 (85.0-98.3) | 33.8 (30.4-37.3) | 11.2 (8.7-14.1) | 98.4 (96.0-99.6) | 38.7 (35.3-42.2) | 1.42 (1.31-1.54) | 0.18 (0.07-0.47) |
| ≥3 | 467 (58.7) | 90.8 (81.0-96.5) | 44.2 (40.5-47.9) | 12.6 (9.8-16.0) | 98.2 (96.1-99.3) | 48.0 (44.5-51.5) | 1.63 (1.47-1.80) | 0.21 (0.10-0.45) |
| ≥4 | 401 (50.4) | 89.2 (79.1-95.6) | 53.1 (49.4-56.7) | 14.5 (11.2-18.3) | 98.2 (96.4-99.3) | 56.0 (52.5-59.5) | 1.90 (1.70-2.13) | 0.20 (0.10-0.41) |
| ≥5 | 336 (42.2) | 83.1 (71.7-91.2) | 61.4 (57.8-65.0) | 16.1 (12.3-20.4) | 97.6 (95.8-98.8) | 63.2 (59.7-66.5) | 2.15 (1.87-2.48) | 0.28(0.16-0.47) |
| ≥6 | 248 (31.2) | 67.7 (54.9-78.8) | 72.1 (68.7-75.3) | 17.7 (13.2-23.1) | 96.2 (94.2-97.6) | 71.7 (68.5-74.8) | 2.43 (1.98-2.98) | 0.45 (0.31-0.64) |
| ≥7 | 171 (21.5) | 61.5 (48.6-73.3) | 82.1 (79.1-84.8) | 23.4 (17.3-30.5) | 96.0 (94.2-97.4) | 80.4 (77.5-83.1) | 3.43 (2.68-4.40) | 0.47 (0.34-0.64) |
| ≥8 | 147 (18.5) | 55.4 (42.5-67.7) | 84.8(82.0-87.3) | 24.5 (17.8-32.3) | 95.5 (93.6-97.0) | 82.4 (79.6-85.0) | 3.65 (2.76-4.81) | 0.53 (0.40-0.69) |
| ≥9 | 79 (9.9) | 29.2 (18.6-41.8) | 91.8 (89.6-93.7) | 24.1 (15.1-35.0) | 93.6 (91.5-95.3) | 86.7 (84.1-89.0) | 3.56 (2.27-5.58) | 0.77 (0.66-0.90) |
| ≥10 | 57 (7.2) | 23.1 (13.5-35.2) | 94.3 (92.3-95.8) | 26.3 (15.5-39.7) | 93.2 (91.2-94.9) | 88.4 (86.0-90.6) | 4.02 (2.36-6.84) | 0.82 (0.71-0.93) |
| ≥11 | 31 (4.0) | 15.4 (7.6-26.5) | 97.1 (95.6-98.2) | 32.3 (16.7-51.4) | 92.8 (90.7-94.5) | 90.5 (88.2-92.4) | 5.36 (2.64-10.88) | 0.87 (0.78-0.97) |
| ≥12 | 24 (3.0) | 13.8 (6.5-24.7) | 97.9 (96.6-98.8) | 37.5 (18.8-59.4) | 92.7 (90.7-94.5) | 91.1 (88.9-93.0) | 6.75 (3.07-14.82) | 0.88 (0.80-0.97) |
| ≥13 | 15 (1.9) | 10.8 (4.4-20.9) | 98.9 (97.9-99.5) | 46.7 (21.3-73.4) | 92.6 (90.5-94.3) | 91.7 (89.6-93.5) | 9.84 (3.69-26.28) | 0.90 (0.83-0.98) |
| ≥14 | 5 (0.6) | 4.6 (1.0-12.9) | 99.7 (99.0-100.0) | 60.0 (14.7-94.7) | 92.2 (90.1-93.9) | 92.0 (89.8-93.8) | 16.87 (2.87-99.15) | 0.96 (0.91-1.01) |
| ≥15 | 4 (0.5) | 3.1 (0.4-10.7) | 99.7 (99.0-100.0) | 50.0 (6.8-93.2) | 92.0 (89.9-93.8) | 91.8 (89.7-93.6) | 11.25 (1.61-78.53) | 0.97 (0.93-1.02) |
| ≥16 | 3 (0.4) | 3.1 (0.4-10.7) | 99.9 (99.2-100.0) | 66.7 (9.4-99.2) | 92.1 (89.9-93.8) | 92.0 (89.8-93.8) | 22.49 (2.07-244.74) | 0.97 (0.93-1.01) |
| ≥18 | 1 (0.1) | 1.5 (0.0-8.3) | 100.0 (99.5-100.0) | 100.0 (2.5-100.0) | 91.9 (89.8-93.7) | 92.0 (89.8-93.8) | Inf (NaN. Inf) | 0.98(0.96-1.01) |
| No., Number; Sensitivity, SE; Confidence Interval, CI; Specificity, SP; Positive Predictive Value, PPV; Negative Predictive Value, NPV; Accuracy, AC; Positive Likelihood Ratio,  PLR; Negative Likelihood Ratio, NLR; Not a Number, NaN; Infinity, Inf. | | | | | | | | |

**Figure S2.** Receiver operating characteristic curve for 30-day mortality based on the *E. coli* score in the subgroup of patients with extended-spectrum β-lactamase production.


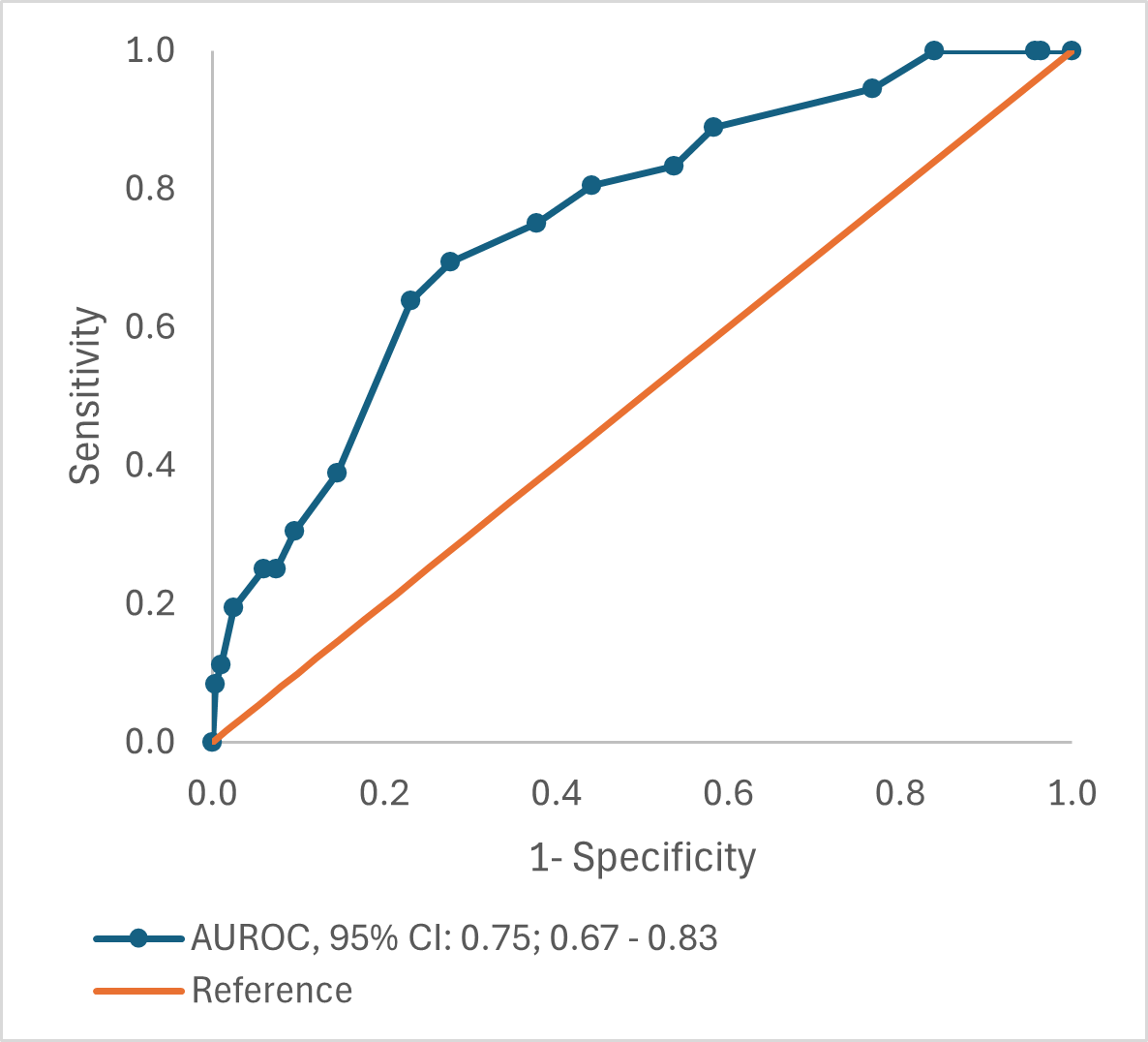


**Figure S3.** Receiver operating curve for 30-day mortality of the gram-negative bacteria predictive model in our database

**
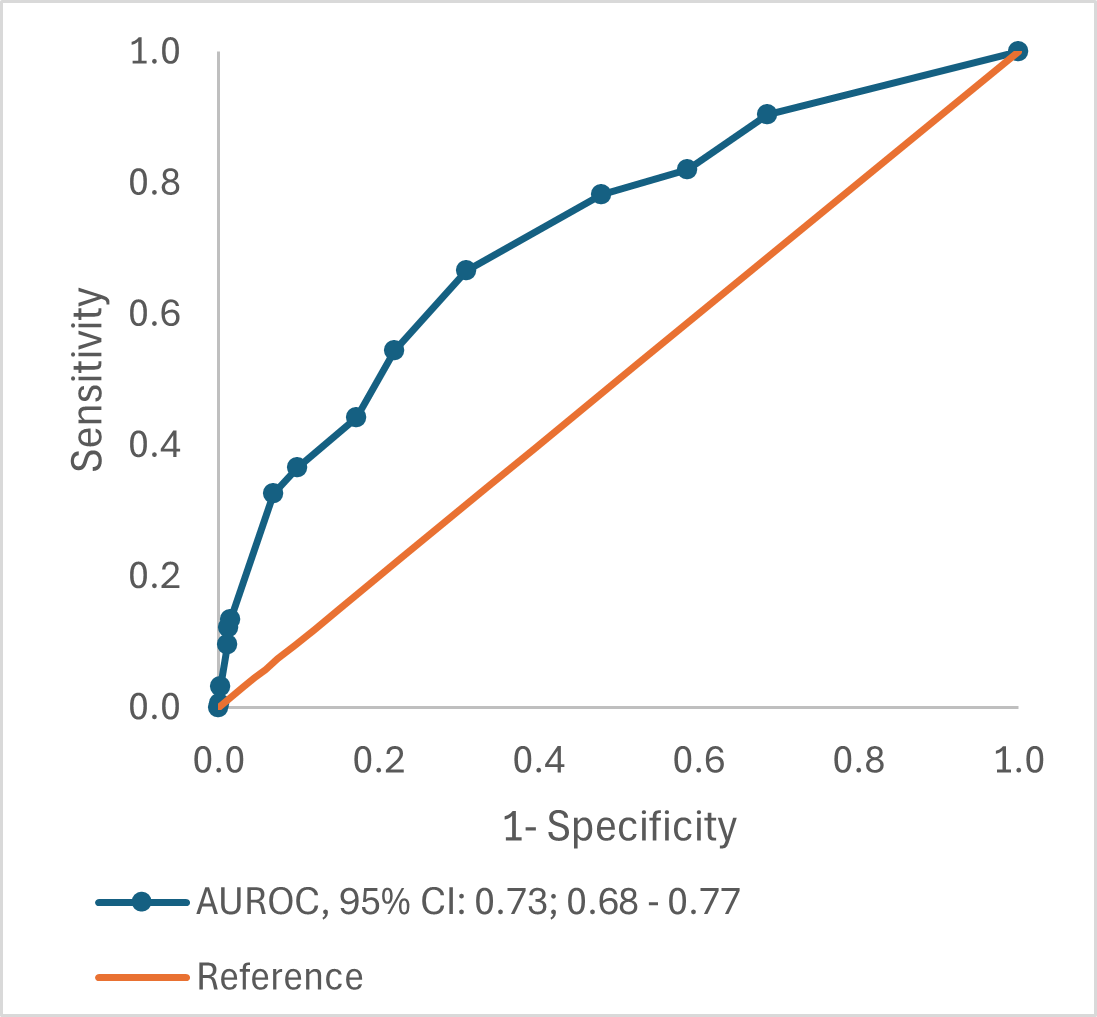
**

**Figure S4**. Receiver operating curve for 30-day mortality of PROBAC score in our database.


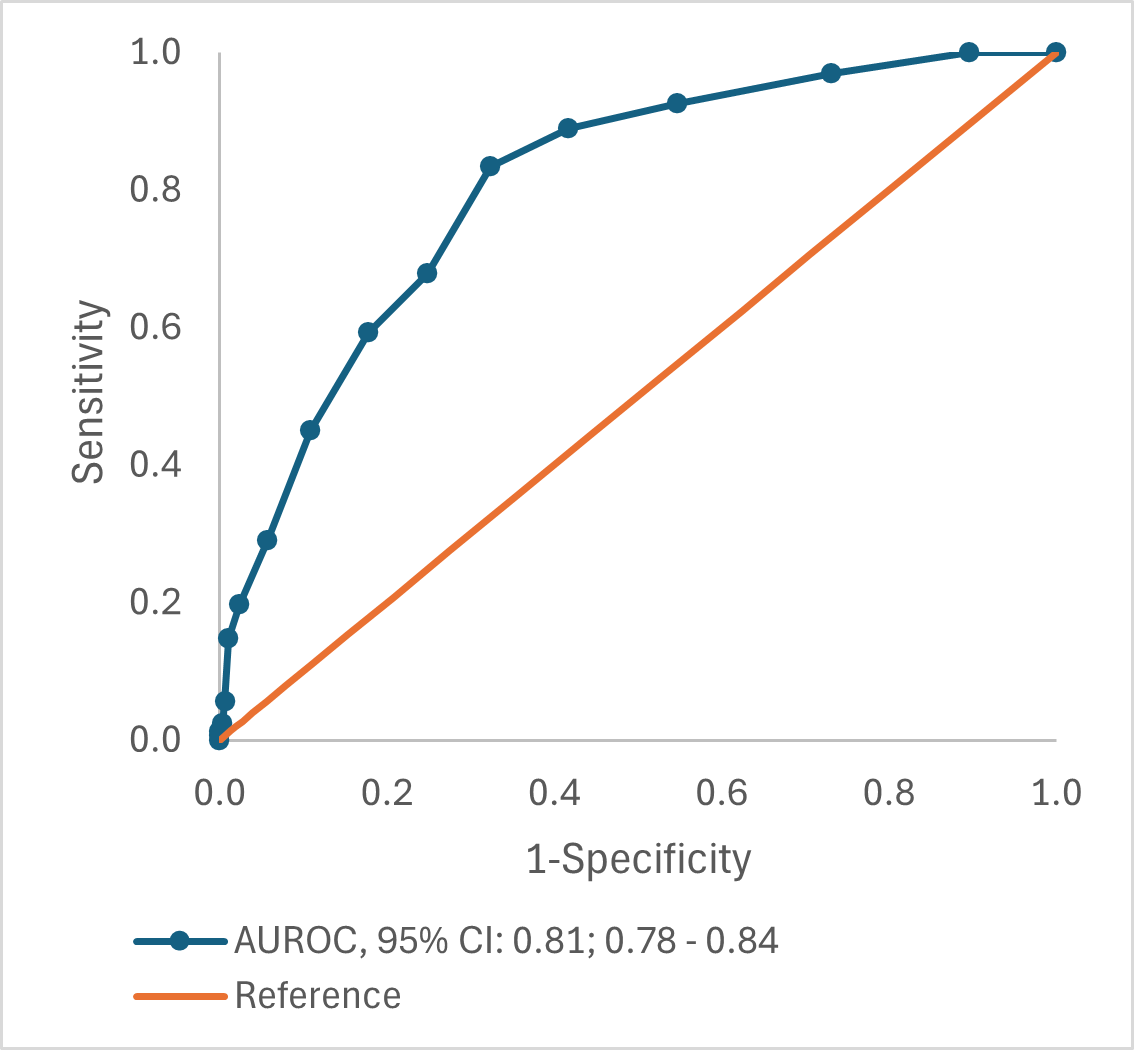


**Figure S5.** Receiver operating curve for 30-day mortality of the Pitt score in our database.


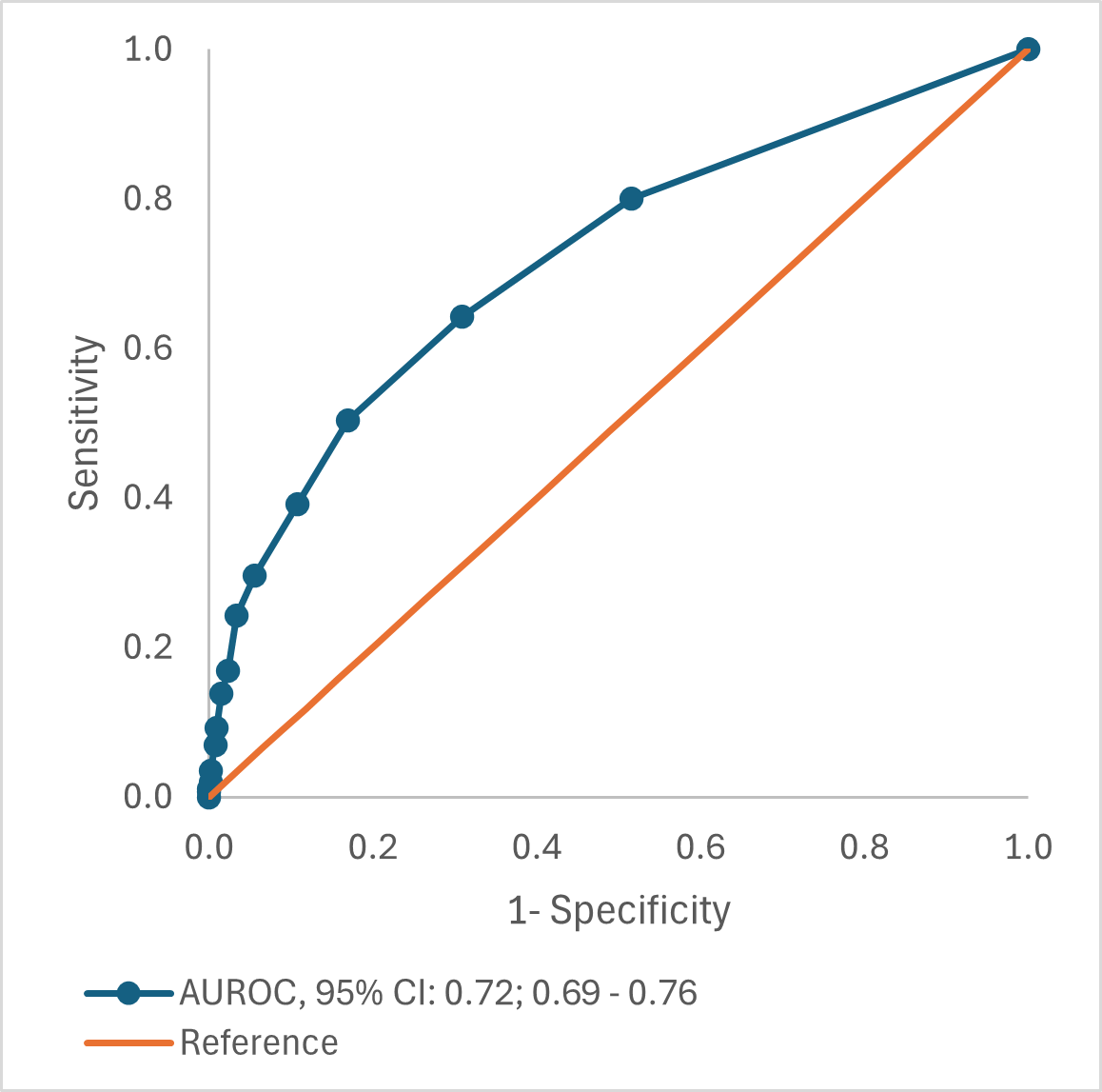


**Figure S6.** Receiver operating curve for 30-day mortality of the SOFA score in our database.


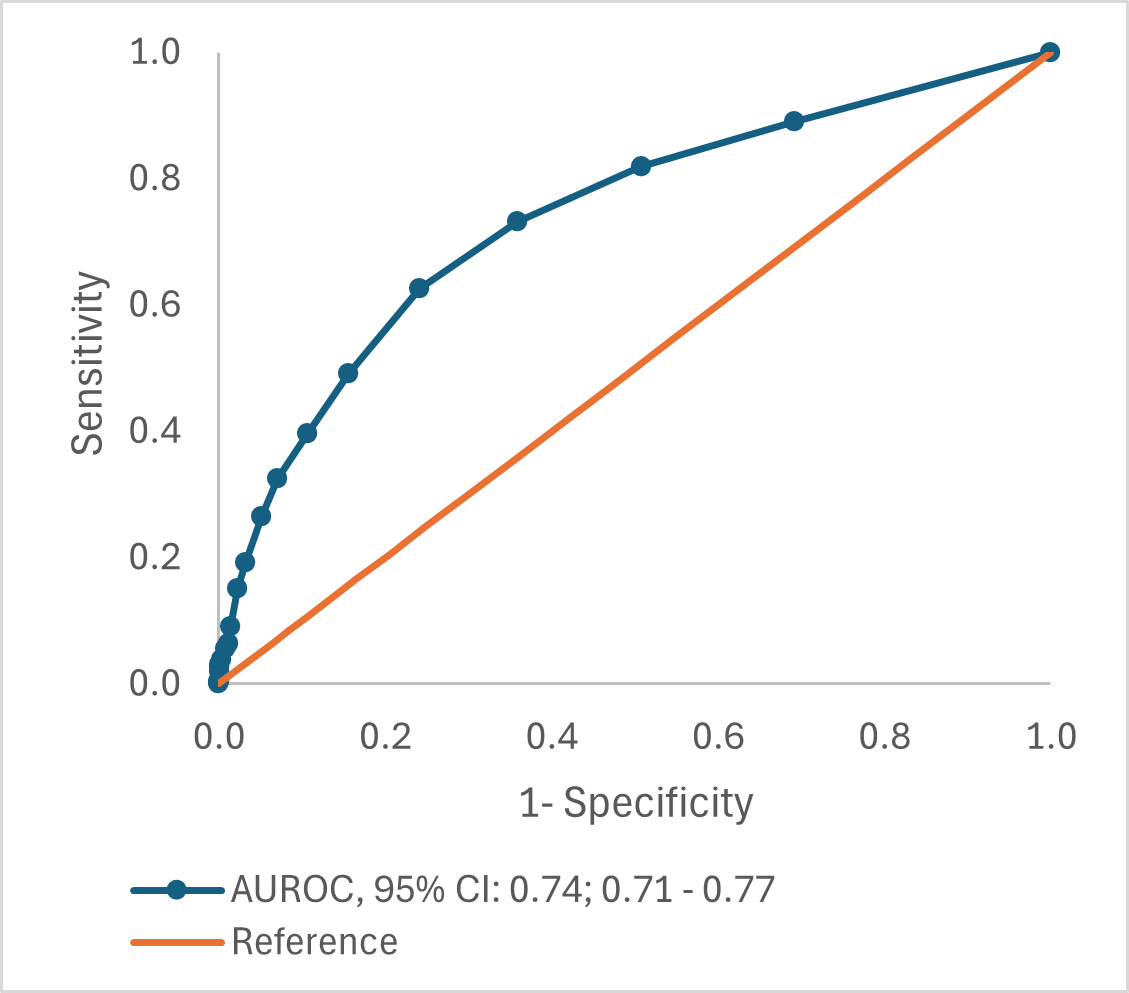


*Legend: Sequential Organ Failure Assessment, SOFA*

**References**

1.Collins GS, Moons KGM, Dhiman P, et al. TRIPOD+AI statement: updated guidance for reporting clinical prediction models that use regression or machine learning methods. *BMJ.* 2024 Apr 16;385:e078378. <https://doi.org/10.1136/bmj-2023-078378>**.**
